# Set-shifting and task-switching make differential contributions to divergent thinking in adolescence

**DOI:** 10.1186/s40359-026-04584-5

**Published:** 2026-04-28

**Authors:** Nastassja L. Fischer, Kastoori Kalaivanan, Ke Tong, Ryutaro Uchiyama, Phillis W.L. Fu, Timothy Lee, Victoria Leong, S.H. Annabel Chen, Trevor W. Robbins, Barbara J. Sahakian, Peter Seow, Chew Lee Teo, David Hung, Michelle R. Ellefson

**Affiliations:** 1 Centre for Research and Development in Learning, Nanyang Technological University, Singapore, Singapore; 2 Centre for Research in Pedagogy and Practice, National Institute of Education, Nanyang Technological University, Singapore, Singapore; 3 Science of Learning in Education Centre, National Institute of Education, Nanyang Technological University, Singapore, Singapore; 4 Humanities, Arts and Social Sciences Cluster, Singapore University of Technology and Design, Singapore, Singapore; 5 Independent Researcher, Singapore, Singapore; 6 Psychology, School of Social Sciences, Nanyang Technological University, Singapore, Singapore; 7 Department of Psychology, University of Cambridge, Cambridge, UK; 8 Department of Psychiatry, University of Cambridge, Cambridge, UK; 9 Faculty of Education, University of Cambridge, Cambridge, UK

**Keywords:** Cognitive flexibility, Cognitive shifting, Task-switching, Set-shifting, Creativity, Divergent thinking, Adolescence

## Abstract

**Background:**

Cognitive flexibility, as the capacity to adaptively shift between mental sets, comprises multiple subprocesses, including set-shifting and task-switching, and goes through changes in capacity throughout development. Divergent thinking, a core aspect of human creativity, also goes through substantial development during adolescence, a period marked by rapid refinement of executive functions. Despite their developmental interdependence, the distinct contributions of cognitive flexibility subcomponents to divergent thinking during adolescence remain insufficiently understood.

**Methods:**

We examined cognitive flexibility and divergent thinking in a large sample of adolescents (*N* = 344). Participants completed established cognitive flexibility measures (e.g., Wisconsin Card Sorting Task; Task-Set Switching), additional cognitive tasks (i.e., working memory, inhibitory control and abstract reasoning) and the Alternate Uses Task to assess fluency and originality in divergent thinking.

**Results:**

Findings provide novel evidence that cognitive flexibility subprocesses differentially contribute to divergent thinking. Specifically, set-shifting, as indexed by Wisconsin Card Sorting Task performance, was a positive predictor of both fluency (i.e., number of ideas generated) and originality (i.e., creative quality of those ideas). In contrast, task-switching performance predicted lower originality, suggesting that rapid, cue-driven shifts may impede the deeper idea exploration required for producing novel responses. This dissociation suggest that cognitive flexibility is not a unitary construct; rather, its distinct components exert separable and, at times, opposing influences on divergent thinking.

**Conclusions:**

These results underscore the importance of distinguishing cognitive flexibility subprocesses when understanding the cognitive mechanisms underlying creativity. The findings carry educational implications, suggesting that fostering strategic, self-guided flexibility, while tempering overly rapid shifting, may support adolescents’ capacity to generate original solutions to complex problems.

**Supplementary Information:**

The online version contains supplementary material available at 10.1186/s40359-026-04584-5.

## Background

Human creativity can be characterized as the adaptive and open-ended evolution of ideas over time [1]. It is an important topic at both societal and individual levels. At the societal level, creativity may lead to important breakthroughs and generate new scientific discoveries, artistic artifacts, technological inventions, and innovation. These, in turn, have substantial economic implications given their potential to result in the development of new products, patents and jobs [2]. At the individual level, creativity may allow someone to come up with original solutions to unexpected problems, whether at school, work, or in daily life.

Despite its importance throughout history, defining creativity and identifying the factors contributing to it remains challenging. Recently, Green et al. [3] reviewed models characterizing creativity both as a product and a process. As a product, the investigation of creativity mostly focuses on the capacity to generate significant creative outputs (e.g., creative achievements; [4–6]). As a process, creativity can be understood as a set of mental events that result in achieving a state of internal attention, which is constrained by a generative goal, where *internal attention* involves searching mental representations of stored ideas, the *generative goal* represents a concept not yet encoded in mental schemas, and *constraints* (e.g., cognitive capacities) guide associations between ideas to form new concepts [3]. This process-based view of creativity is supported by cognitive processes, such as working memory, inhibitory control, cognitive flexibility, and general cognitive abilities [7].

Divergent thinking, the ability of producing diverse ideas or solutions to solve a specific problem [8], is a key cognitive process underlying creativity and has long been recognized as a central component of creative potential [9–13]. It is distinct from, though related to, convergent thinking, which involves integrating multiple seemingly unrelated concepts to find a solution for a given problem [14]. Therefore, while creativity encompasses the broader capacity to generate novel and useful ideas or products, divergent thinking specifically refers to the cognitive process of generating multiple diverse ideas.

Research shows that although cognitive processes underlying divergent thinking may be complex, a better understanding of its mechanisms could facilitate teaching divergent thinking abilities from early ages [15]. Increasing evidence highlights adolescence as a period for the development of creative abilities, particularly divergent thinking, which seems to support adaptive decision making, flexible learning from feedback, and effective functioning in complex social situations up to adulthood years [16–18]. Divergent thinking also underpins other essential abilities that become increasingly important throughout the school years, such as curiosity [19, 20] and self-regulated learning [21]. Furthermore, divergent thinking has been positively associated with academic achievement in varied education stages [22]. Identifying the individual factors (e.g., cognitive flexibility abilities) that influence divergent thinking during adolescence is especially critical, as this period represents a key window for cultivating creative development [13, 16, 17].

This study examines the *process* aspect of creative idea generation in adolescence, specifically investigating how cognitive flexibility and its distinct subcomponents (i.e., set-shifting and task-switching) contribute to divergent thinking performance. To provide a background about the relevance of cognitive flexibility to divergent thinking in adolescence, in the following sections, we will introduce some theoretical foundations for the study by: (1) defining cognitive flexibility and its measurement, (2) reviewing cognitive flexibility development during adolescence, and (3) synthesizing previous findings on the relationship between cognitive flexibility and divergent thinking. Our aim is to elucidate how adolescence serves as a critical period for developing flexible thinking abilities foundational to divergent thinking.

### Cognitive flexibility – definition and measurement

Although there is no universal definition, *cognitive flexibility* is broadly conceptualised as the ability to adaptively switch between cognitive tasks or mental sets in response to changing demands [23, 24]. It interacts dynamically with other cognitive processes such as working memory, inhibitory control, and abstract reasoning to support flexible goal-directed behaviour (i.e., the ability to act with the intention of achieving a specific outcome [25]), playing critical roles in shaping life outcomes, such as academic achievement, social interactions, and mindsets [23, 26, 27].

Commonly used cognitive flexibility assessments include the Wisconsin Card Sorting Task [28, 29], Task-Set Switching paradigms [30], the Trail Making Test A and B [31, 32], and the CANTAB Intra- and Extra-Dimensional Shift Task [33]. Because these tasks engage partially distinct abilities that fall under the broad umbrella of cognitive flexibility construct, some authors have argued for more fine-grained frameworks. Specifically, cognitive flexibility may be decomposed into *set-shifting* versus *task-switching* abilities [34], or *cognitive shifting* versus *contingency-based switching* subcomponents [35]. These classifications emphasize the non-unitary nature of the cognitive flexibility construct.

More specifically, set-shifting refers to the ability to shift between different rules or stimulus-response mapping within a same task (i.e., set of instructions that defines successful performance), guided by feedback [34]. For example, in cognitive flexibility tasks requiring set-shifting (e.g., Wisconsin Card Sorting and Intra-Extra Dimensional Shift Tasks [34, 35]), rather than alternating between distinct instructions, the participant needs to shift their response according to different features of a stimuli to complete an unchanged task demand (e.g., to sort cards according to a reference stimulus using a rule that it is not explicitly stated).

In contrast, task-switching involves alternating between two or more tasks, typically cued explicitly (e.g., to categorize a stimuli as a vowel/consonant or odd/even, depending on their position at the computer screen), and relies on rapid reconfiguration of task parameters (e.g., Trail Making Test, cued task-switching paradigms; [34, 36]). Both set-shifting and task-switching fall under the broader cognitive shifting subcomponent as conceptualized by Parr et al. [35], which encompasses higher-order processes, that allow adapting attention, strategies, or behavioural outputs following a switch in task goal, rules, or mental set. These higher-order processes are interpreted by some as being part of cognitive control [37] or executive function [38] abilities, with both terms being defined as a set of capacities that enable someone to guide thoughts and actions in line with desired outcomes. Overall, according to previous studies [35, 39, 40], it is hypothesized that cognitive shifting should support the balance between idea generation (i.e., memory retrieval, mind wandering, semantic integration, associative thoughts, and mental imagery) and idea selection (i.e., selection of the most useful and innovative ideas), which are foundational for optimal divergent thinking performance.

### Cognitive flexibility during adolescence

Cognitive flexibility development begins in infancy and continues through early childhood and adolescence into adulthood [41–43]. The growing evidence linking cognitive flexibility to age-related changes in learning underscores its importance during childhood and adolescence, when individuals are developing the ability to adapt to new rules, learn from feedback, and adjust their behaviour across different environmental contexts [34, 36, 44]. For instance, initial evidence suggests that cognitive flexibility is linked to better reading and numeracy skills in childhood [45, 46] and adolescence [47], higher resilience in adulthood [48], and indexes of creativity in children, adolescents and adults [49, 50]. Additionally, the significant socio-emotional changes during adolescence (e.g., changes in social environment and emotional regulation; [16, 17]) underscore the importance of studying cognitive flexibility to understand how adolescents can navigate uncertainties effectively. Therefore, a growing focus on operationalizing cognitive flexibility helps to elucidate how it is linked to positive outcomes across the lifespan.

### Contribution of cognitive flexibility to divergent thinking during adolescence

Adolescence represents a key stage in the development of divergent thinking [16, 17]. Divergent thinking abilities have been observed from early childhood, with a developmental pattern being consistent with an overall upward trend punctuated by periods of irregular improvements observed throughout childhood and adolescence [10]. Said-Metwaly et al. [51]’s meta-analysis identified that some of the observed non-linear trends in the divergent thinking development may happen due to some moderating factors, such as task type, content domain, educational stage, and participants’ individual differences (e.g., sex). However, it remains unclear how relevant individual factors, such as cognitive flexibility, influence divergent thinking progress throughout the years [52].

Cognitive flexibility is considered an important facilitator of divergent thinking [7, 23, 53–55], although the precise mechanisms behind it remain unclear. Palmiero et al. [7] identified only eight studies directly exploring this relationship, with seven of them involving adults, and just one [49] focusing on children and adolescents. In adult populations, studies examining cognitive flexibility and divergent thinking show mixed outcomes, with some reporting significant positive associations and others finding no reliable connections between the two variables [7].

The mixed findings concerning the influence of cognitive flexibility on divergent thinking likely reflect variation in how cognitive flexibility is assessed across studies, including overlap with tasks assessing divergent thinking or the narrow focus on pure perceptual/attentional shifting (i.e., task-switching) tasks [7]. For example, existing findings suggest that measures capturing spontaneous flexibility (i.e., the ability to freely generate different categories of responses; [56]) during late childhood and adolescence may be associated with divergent thinking [49, 57], whereas task-switching performance has not shown reliable associations with creativity outcomes [57, 58]. However, the use of spontaneous flexibility as a measure of cognitive flexibility, as suggested by Marron & Faust [56] may potentially conflate both predictor and outcome variables, when the latter include creativity indicators [7]. Meanwhile, other studies usually rely on a single task [58, 59] or on multiple tasks within a same cognitive flexibility specific ability, such as task-switching [60], to entail cognitive flexibility. This may lead to an incomplete representation of the abilities included in this construct. Taken together, literature points to the need for a broader and more comprehensive approach that assesses both set-shifting and task-switching abilities as distinct subcomponents of cognitive flexibility to clarify its contribution to divergent thinking during adolescence.

### The current study

This study investigates how cognitive flexibility and its set-shifting and task-switching subcomponents uniquely contribute to divergent thinking during adolescence, as outlined in our pre-registration document [https://osf.io/md4tv, hypothesis 5: “Cognitive flexibility latent variable(s) will be associated with at least one primary outcome variable (creativity, literacy, and numeracy).”]. Following the pre-registered plan, we conducted correlations, confirmatory factor analyses, and hierarchical regression linear mixed modelling to: (1) examine the relationships between divergent thinking, cognitive flexibility, other cognitive abilities; and (2) explore the dissociation between cognitive flexibility and additional executive function components in their influence on divergent thinking in adolescents.

We operationalise divergent thinking by extracting measures of fluency and originality from the Alternate Uses Task, which is the most used divergent thinking task in the literature, and has demonstrated to yield reliable estimation of domain-general divergent thinking capacity [61]. Fluency (i.e., number of unusual ideas) and originality (i.e., how creative and unusual these ideas are) are often used measures that capture the quantitative and qualitative aspects of idea generation in divergent thinking [62].

In parallel, we operationalise cognitive flexibility through four tasks identified by previous literature [35, 37] as assessing the distinct subcomponents of cognitive shifting [36]. Set-shifting is measured using the Wisconsin Card Sorting (Wisconsin; [28]); and the Intra-Extra Dimensional Shift tasks (Intra Extra Shift; [63, 64]). Task-switching abilities are measured using the Trail-Making task A and B (Trails; [32]), and Task Set-Switching (Switching; [65]). We note that while other tasks, such as the colour-shape and number-letter switching paradigms [66, 67], are also commonly used measures of cognitive flexibility; they represent variants of cued task-set switching already captured by our task-switching paradigm. To ensure participant engagement and good data quality, and to avoid lengthy experimental procedures, we prioritized breadth across the cognitive flexibility subcomponents over depth within a single subtype. Our chosen tasks also demonstrated consistent neural correlates across different studies [34–36, 68–70], reinforcing their validity as measures of core cognitive flexibility processes.

When addressing our key research questions, we recognize that cognitive flexibility does not operate independently [34, 66]. Other cognitive abilities including inhibitory control, working memory and abstract reasoning are also critical for higher-order cognitive processes like divergent thinking [7, 22, 60, 71–74]. More specifically, working memory capacity tends to show a positive relationship with divergent thinking, especially by increasing the number of ideas (i.e., fluency) rather than their originality [7, 75]. Meanwhile, findings on inhibitory control are mixed, with some studies reporting that stronger inhibition (i.e., better suppression of dominant responses) is associated with higher creativity, whereas other work links lower inhibition (i.e., a more disinhibited attentional state) to greater idea fluency and originality [7, 76]. Abstract reasoning has also shown a modest positive correlation with divergent thinking performance, often overlapping with cognitive processes like inhibitory control or working memory (e.g., when the influence of abstract reasoning is statistically controlled, the unique link between working memory and divergent thinking tends to disappear; [7]). In summary, these findings highlight that working memory, inhibitory control, and abstract reasoning abilities contribute to divergent thinking and may interact with the effects exerted by cognitive flexibility subcomponents. Therefore, we include these cognitive abilities as covariates in our analyses to understand the unique contribution of cognitive flexibility to divergent thinking during adolescence.

## Method

### Participants

Our total sample included 344 Singaporean secondary school students (*n* = 191 girls, *n* = 153 boys; X_age_ = 13.11, SD_age_ = ± 0.53 years). We included participants having more than 20% of the trial-level data for the cognitive variables that were not excluded due to missing data or performance-based exclusion of trials. Moreover, all our participants completed more than 80% of the entire task battery. This sample size and participant inclusion criteria align with our pre-registered power calculation that followed a frequentist statistical approach (details available at https://osf.io/xf78p/), although a Bayesian sequential stopping rule was also preregistered. This happened as our recruitment procedures enabled us to closely reach the predicted target number size derived from the frequentist power analysis. The ethnicities of the participants were: *n* = 205 Singaporean Chinese (60%), *n* = 58 Singaporean Indian (17%), *n* = 47 Singaporean Malay (14%), and *n* = 23 participants from other ethnic origins (7%), and *n* = 11 did not declare ethnicity (3%). All participants were enrolled in school, with English being the main language taught and spoken in schools in Singapore [77].

Additional descriptive analysis with participants’ family socioeconomic status (i.e., parental educational background), and age from our final sample is depicted in the Results section (Tables 1 and 2). Students or parents answered about parental educational level by answering the question: “What is the highest level of education your father has completed or the highest degree they have received?”. The same phrasing was implemented when asking about the mother’s latest educational attainment. When parents answered the questions, they were instructed that the answer should reflect their own educational background. The possible responses they could assign were: (1) No schooling, (2) Primary school, (3) Secondary school, (4) Certificate/Diploma (i.e., Professional certificate), (5) Undergraduate/Bachelor’s Degree, (6) Master’s degree, (7) PhD/Doctoral degree, (8) Other. If they indicated the last option, they could detail which was the latest degree they had that was not included in the response list.

We recruited participants through local schools and paid advertisements on social media. Both parents and students consented to the study by signing and submitting informed consent forms beforehand. The Institutional Review Board at Nanyang Technological University (IRB-2020-06-030) conducted the ethical review. All procedures were in accordance with the Declaration of Helsinki.

### Procedures

We collected data using two assessment modes. In schools, we used a grouped assessment approach in classrooms, following Obradović et al. [78]. At our university lab facilities, we used an individual assessment mode with an experimenter-to-participant ratio of either 1:1 or 1:2 during each session. Across both modes, participants completed tasks using portable computers with a wired mouse, with breaks between cognitive tasks to mitigate fatigue. The maximum duration of the complete pre-registered experimental procedures (https://osf.io/md4tv/overview) was up to 4 h for the grouped assessment mode, and up to 3 h for the individualized assessment, both including breaks. This difference of duration time between the assessment modes happened to accommodate our procedures to the school’s schedule in the grouped setting. These two assessment modes allowed greater scalability and enhanced sample representativity.

We performed Fisher’s *z*-transformation tests to verify whether correlations between the variables of interest differ significantly between the assessment modes (see Supplementary Information; Table S1).

### Measures

Participants performed a battery of computerised tasks evaluating divergent thinking, cognitive flexibility, working memory, inhibitory control, and abstract reasoning. The task order was randomized to prevent performance biases due to order effects.

For divergent thinking, participants completed a computerized version of the Alternate Uses Task (Alternate Uses; [8]) administered on the Inquisit platform [79]. In the Alternative Uses, participants were required to generate as many non-typical, uncommon and creative possible uses of common household items: paperclip, shoe and nail. For each item, participants were given 3 min to generate responses. The total duration time for participants to complete this task was approximately 15 min (including instruction time and practice trial). We focused our analysis in extracting two key indicators of divergent thinking: fluency and originality. Fluency was scored as the total sum of different unusual and creative responses at each item, averaged across all the items and raters. Originality was coded using the subjective scoring method [80] with coders evaluating how creative ideas were, on a range of 0 to 4 points. The maximum score was given to “very creative” responses when they were considered as novel, unusual, or original, and appropriate, effective, clever, interesting, humorous, or surprising [61]. All the scores were performed by two independent raters. Following Saretzki et al. [61]’s findings, we chose the Max-3 (i.e., average across the three responses that were the most highly rated as original for each of the items) and Snapshot (i.e., originality rating of the entire set of responses for each of the items; [80]) scores to entail originality in the Alternate Uses, since these were the ones with the highest validity evidence reported in the literature. Since we aim to identify the best indicator of divergent thinking in our sample of adolescents, we included both measures in our analysis.

Interrater reliability for the Alternate Uses was strong across the fluency and the two originality scoring approaches. For fluency, reliability (two-way random, absolute agreement, 2 raters; ICC (3,2)) was good to excellent for all the three items, ranging from 0.838 to 0.951. For originality, Max-3 scoring yielded good to excellent reliability, with ICC (3,2) values in between 0.774 and 0.804. Similarly, Snapshot scoring indicated excellent agreement between raters across all three items (i.e., ICC (3,2) from 0.836 to 0.864). Given that the scoring methods show consistent ICC ratings, their values were averaged across the different coders and items to index participants’ performance in the fluency and originality categories. Internal-consistency analyses indicated that the AUT fluency scores have acceptable reliability (Cronbach α = 0.842; McDonald’s ω = 0.849). For the originality ratings, internal consistency varied by scoring method showing acceptable overall results, with the Max-3 originality scores demonstrating α = 0.733; and ω = 0.757, whereas the Snapshot originality ratings showed reliability levels as α = 0.756; and ω = 0.757.

To measure cognitive flexibility, we used four tasks in our study: Wisconsin Card Sorting Task (Wisconsin; [28]); Intra-Extra Dimensional Shift (Intra Extra Shift; [63, 64]), Trail-Making Task A and B (Trails; [32]), and Task Set-Switching (Switching; [65]). These tasks were chosen since they are all identified by previous literature as entailing task-switching (e.g., Trails and Switching) and set-shifting (e.g., Wisconsin and Intra Extra Shift) abilities [34, 36]. These are used here to indicate the different facets of someone’s capacity to switch reactively in response to deterministic task-response rules, being also interpreted as part of the cognitive shifting subcomponent contained in the broad cognitive flexibility construct [35].

A computerized version of Wisconsin [28] was administered on the Inquisit platform [79]. In this task, participants were shown a stimulus card and four decks of cards. They had to figure out which deck the stimulus card should be sorted into. The sorting rule could be according to color, shape or number, and unbeknown to the participants, it changed when 10 consecutive correct choices were made. Because participants were not explicitly informed when the rule would change, they had to figure out the rule by themselves in a self-guided way, through trial-level feedback. The full task had a total of 128 trials, with an approximate duration of 8 min (including instruction time). In our study, we used the reversed score of the number of preservative errors as a performance indicator to indicate that higher the values in Wisconsin, the better. Perseverative errors were computed by the Inquisit platform [79] task script, with practice trials being excluded by design. Trials were dynamically classified as valid according to the active sorting rule and feedback, and perseverative errors were computed only when participants successfully learned a sorting rule. Reliability (split-half estimate) was *r* = .930 and the Spearman–Brown–corrected reliability was 0.964.

The Intra–Extra Dimensional Shift task (CANTAB^®^; [62, 63]) required participants to learn stimulus–response rules through feedback (“correct” or “wrong”). Across the task, the stimulus that yields a correct response changes, and task difficulty increases from simple single-dimension discriminations (e.g., shape) to compound stimuli involving both shapes and lines. Participants begin with an intra-dimensional shift stage and can progress up to Stage 9 (i.e., second reversal learning stage), during which the rule shifts from intra-dimensional (e.g., change in the correct shape) to extra-dimensional (i.e., change in the stimulus set that is correct) in a second time. To progress across this task, participants are required to have six consecutive correct responses per stage, and thus total trials vary with performance. In our sample, participants completed an average of 81 trials, with an approximated completion time of 11 min (including instruction time). Performance was operationalised as the reversed score of the total number of errors at Stage 8 (i.e., first reversal learning stage), with higher scores being interpreted as better performance in this task. The total number of errors at Stage 8 were derived from the output table generated by the Cambridge Cognition (https://cambridgecognition.com/) platform, with data cleaning done using their proprietary algorithms. Reliability for the Intra–Extra Shift score exhibited a split-half estimate of *r* = .863 and a Spearman–Brown–corrected reliability of 0.926.

Additionally, we used a computerized version of Trails-Making Task A and B (Trails; [33]) on the Inquisit platform [79] that consisted of two parts: part A, where participants were shown numbers in circles and were tasked to connect the circles in order as fast as they could; and part B, where the circles contained numbers and letters that needed to be connected in crescent order (e.g., 1-A-2-B). The average completion time for this task was approximately 6 min, including instruction time. Trails performance measures (i.e., time to complete parts A and B) were derived from Inquisit generated summary files. Because the task enforces correctness during execution, such that errors delay progression but are not excluded, metrics reflect overall task completion performance. For Trails, similarly to what we implemented for the Wisconsin and Intra–Extra Shift tasks, we used the reversed score of the difference in time to complete part B compared to part A (B-A), with higher score representing better performance in this task. Previous research indicated the difference in time to complete part B compared to part A as the most reliable measure of cognitive flexibility abilities using Trails in developmental samples [31]. Internal reliability metrics (e.g., split-half) are not applicable to the Trails task because each set (A and B) consists of a single administration (i.e., it is not possible to compute item-level variance). Nevertheless, past research [31] has shown fair to good test-retest reliability at the 1-month interval in 9 to 12-year-olds (intraclass correlation coefficient; ICCs > 0.53) for this task, which reinforces its utility as an indicator of cognitive flexibility.

Finally, we implemented a Task-Set Switching Task (Switching; [65]) on the Gorilla platform (www.gorilla.sc). There, participants were shown a cue (i.e., either a circle or a square) in which a number was paired up with a letter (e.g., 8 A). Depending on the cue, participants could respond differently: if a circle was shown, participants should respond to the letter in the circle by pressing the left or right keyboard button, to indicate where the letter was (i.e., at the left or right side of the screen). If the cue was a square, participants should respond to the number and indicate whether the number was. Sanity checks were implemented to ensure participants understood the rules before the start of the actual task. On average, participants completed 190 trials. This task had an approximated duration of 17 min, including instruction time. Given that data were collected using both individual and group-based assessment modes, we focused on accuracy-based switch costs to entail participants’ performance. We did so following practices implemented in other studies implementing a similar experimental design that involved data collection of cognitive measures in school settings [78, 81]. Accuracy in switch and non-switch trials were computed from all non-timeout trials. Switch costs were computed as the difference in mean accuracy between switch and non-switch trials (switch − non-switch), with larger positive values indicating better performance on task-switching. The split-half reliability of our Switching task was *r* = .221, corresponding to a Spearman–Brown corrected estimate of 0.362.

We also included the following tasks to measure additional cognitive abilities supporting cognitive flexibility: CANTAB Spatial Working Memory [82] to entail working memory, Stroop [83] to measure inhibitory control, and Raven’s Standard Progressive Matrices [84] for abstract reasoning abilities. These tasks were chosen since we wanted to understand the impact of cognitive flexibility abilities on divergent thinking while considering the potential contribution of additional cognitive abilities known to interact with cognitive flexibility [34].

In our study, we administered the CANTAB^®^ Spatial Working Memory task (Working Memory; [82]), where participants were tasked to find a token hidden under boxes, which only appeared once under each box. The number of boxes throughout the task increased once the participants progressed across the task’s levels. The total number of between errors (i.e., number of times a participant revisits a box that has already been found to contain a token) made at all stages was used to indicate performance in the Working Memory task. This performance indicator was derived from the output table generated by the Cambridge Cognition (https://cambridgecognition.com/) platform, with data cleaning done using their proprietary algorithms. Participants took approximately 9 min to complete this task, including instruction time. Internal consistency could not be estimated for this task, as its structure does not provide multiple interchangeable items suitable for psychometric reliability analyses. Although high internal consistency values have been reported in young children (e.g., α ≈ 0.95; [85, 86]), these estimates come from versions of the task that allow such computation and may not be generalizable to the paradigm adopted in the current study.

We also used a computerized version of the Stroop task [83] as implemented on the Inquisit platform [79]. In each trial, one stimulus word was shown to the participants who were required to respond to the colour of the word as fast as possible. In congruent trials, participants saw a word that corresponded to the colour it was printed (e.g., “Red” word in red colour), while in incongruent trials participants saw a colour name that was printed in a different colour (e.g., “Red” word in green colour). Participants were instructed to respond only to the colour of the word and to ignore its meaning. The task had a total of 180 trials, and participants took approximately 10 min to complete the tasks, including instruction time. Accuracy in switch and non-switch trials were computed from all non-timeout trials. We used the switch-cost in accuracy (i.e., accuracy between congruent versus incongruent trials) to indicate performance in this task. In our study, the Stroop task demonstrated a split-half correlation of *r* = .450, with a Spearman–Brown–corrected reliability of 0.620.

Finally, we administered the abbreviated 9-item Standard Ravens Progressive Matrices (Ravens) revised by Bilker et al. [84], as a measure of abstract reasoning and fluid intelligence. Ravens is a multiple-choice test with no time constraints where participants were shown a series of incomplete matrices, and their task is to complete the matrices by selecting one of the four options provided. This abbreviated version was shown to be comparable to its original form [84], and participants took approximately 8 min (including instruction time) to complete it. Performance was computed from valid response entries, by excluding records with missing item codes and duplicate or inconsistent item logs. In our study, we used the total number of correct responses as a performance indicator. Our Ravens task showed the following values of internal consistency: Kuder-Richardson-20, KR-20 = 0.57, ω = 0.57, and Spearman–Brown = 0.61. Item difficulties ranged from 0.13 to 0.82 and discrimination indices were generally satisfactory (median *r* = .31) for our adolescent sample.

### Data analysis plan

We adjusted the pre-registered analysis plan due to logistical constraints and theoretical considerations. First, although Bayesian methods were preregistered as a possible analytical approach, all inferential analyses reported in the present manuscript were conducted using frequentist statistical approaches only. Second, we excluded literacy, and numeracy measures since this analysis was not within the scope of this paper. Third, we shifted from using latent variables as evidence suggests latent executive function factors may be inappropriate for developmental studies due to poor task correlations [87]. Moreover, when running confirmatory factor analysis to verify the suitability of the cognitive flexibility latent variable, we found that although our results demonstrated excellent overall fit to the data (𝜒^2^(2) = 2.026, *p* = .351; CFI = 0.999; TLI = 0.998; SRMR = 0.021; Adjusted RMSEA = 0.006), a closer examination of the measurement model showed mixed results. More specifically, while three indicators (Wisconsin, Intra Extra Shift, and Trails) had significant (i.e., *p* ≤ .05) standardized loadings (𝜆_std_ = 0.61; 0.55; and 0.29; respectively), Switching exhibited a non-significant and very weak loading (𝜆_std_ = 0.02, *p* = .74), suggesting it does not contribute meaningfully to the construct. As the Switching task performance should have an important theoretical contribution to someone’s overall cognitive flexibility abilities, we decided that the tasks implemented here should be better analyzed in separate instead of using a latent variable. In this way, we should be able to investigate how different facets of the cognitive flexibility (shifting) subcomponent (i.e., task-switching and set-shifting abilities) contribute to divergent thinking during adolescence.

Additionally, we did not include the Probabilistic Reversal Learning Task in our current analysis due to its distinct probabilistic nature [35] when compared to the other cognitive flexibility tasks. Its analysis will be included in a future manuscript. We also excluded the self-report questionnaires related to flexible thinking since the focus of the current study is on the cognitive aspects influencing divergent thinking in the Alternate Uses. Their analysis will also be addressed in a separate study. Finally, we excluded research questions unrelated to creativity to align with the scope of this paper.

All metrics analysed here were standardized using z-scores to uniformize effect sizes across different performance indicators. Between participants, we excluded performance indicators from tasks having outliers exceeding 2.5 standard deviations from the mean. The missing data rate for all variables remained below the 20% threshold specified in our preregistration (https://osf.io/xf78p/). An expected 20% attrition rate when implementing cognitive testing to adolescents is well-supported by prior studies using similar samples, with attrition commonly falling in between the 10–25% range depending on the context [88, 89]. For the correlational analysis and regression models we adopted multiple imputation using the predictive mean method as implemented in the *mice* package in *R* [90] for all the variables included in this study.

After imputation, descriptive statistics, bivariate correlations, and regression models were performed to verify the associations of divergent thinking metrics (i.e., fluency and originality) with the cognitive flexibility tasks and the additional measures (Working Memory, Stroop, Ravens, Age, Mother’s Education and Father’s Education). To account for multiple comparisons, we applied Bonferroni correction for the correlation analysis (i.e., significance at *p* ≤ 6.00E-05 for 78 correlations).

To further examine the effect of the different abilities included in the cognitive flexibility construct on divergent thinking during adolescence, hierarchical regression models were conducted, with the cognitive flexibility variables (i.e., Wisconsin, Intra Extra Shift, Trails and Switching), Working Memory, Stroop, and Raven’s as independent variables, and Age, Mother’s Education and Father’s Education as controlling variables. Divergent thinking metrics (Alternate Uses: Fluency, Alternate Uses: Originality [Max-3] and Alternate Uses: Originality [Snapshot]) were included as dependent variables. We incrementally introduced predictors to assess their individual contribution to each dependent variable.

Because participants were nested within two assessment modes (i.e., grouped and individualised), hierarchical regression analyses were conducted using linear mixed-effects modelling with random intercepts for the type of assessment mode (i.e., Setting), which accounts for non-independence of observations while allowing for differences in overall outcome levels between Settings. Marginal R² values for the linear mixed-effects models were computed following the Nakagawa et al. [91] method, which estimates the proportion of variance explained by the fixed effects in mixed-effects models. All coefficient estimates reported in tables and text are based on Restricted Maximum Likelihood fitted models, which provide less biased estimates of variance components and fixed effects in mixed models than maximum likelihood estimation [92]. To evaluate incremental model fit across hierarchical steps, model comparisons were conducted using maximum likelihood estimation, as likelihoods from Restricted Maximum Likelihood fitted models are not comparable when fixed effects differ [92]. Model comparisons were performed using pooled Wald tests (D2 method) across the multiply imputed datasets [93]. All model estimates were pooled across multiple imputed datasets [94]. Statistical significance was considered at *p* ≤ .05.

Finally, to assess multicollinearity among predictors, we computed Variance Inflation Factors (VIFs) for all variables entered in the regression models. We considered an accepted thresholds of VIF value < 5 as indicating no problematic multicollinearity [95].

## Results

### Descriptives statistics and correlation analysis

Descriptive statistics with participants’ family socioeconomic levels (i.e., parental education), age, and cognitive tests, together with the correlation statistics results, are depicted in Tables 1, and 2. A full correlation matrix with the sample’s histograms, r and exact p values can be found in Supplementary Information (Figure S1).

**Table 1:** Family Socioeconomic Status Summary (*N* = 344)

Educational level	Mother’s Education - *N* (%)	Father’s Education - *N* (%)
Primary school	4 (1)	7 (2)
Secondary school	34 (10)	35 (10)
Junior college	16 (5)	9 (3)
Professional certificate	94 (27)	83 (24)
Undergraduate degree	87 (25)	88 (26)
Masters’ degree	48 (14)	59 (17)
Doctoral degree	7 (2)	10 (3)
Not declared	54 (16)	53 (15)

**Table 2:** Descriptive Statistics and Correlations for the Creativity and Cognitive Tasks Variables (N = 344)

Variable	M	SD	1	2	3	4	5	6	7	8	9	10	11	12	13
1. Alternate Uses: Fluency^a^	13.11	0.53	-												
2. Alternate Uses: Originality (Max 3)^b^	1.47	0.66	.66*	-											
3. Alternate Uses: Originality (Snapshot)^c^	1.4	0.72	.57*	.94*	-										
4. Age	13.11	0.53	.20	.34*	.32*	-									
5. Mother's Education	4.37	1.3	.17	.27*	.30*	.12	-								
6. Father's Education	4.47	1.39	.10	.22	.24*	.13	.61*	-							
7. Working Memory^d^	40.46	17.21	.18	.27*	.28*	.22	.18	.17	-						
8. Stroop^e^	-0.12	0.08	-.10	-.07	-.07	.02	-.06	-.09	.10	-					
9. Ravens^f^	4.33	1.9	.17	.29*	.29*	.17	.20	.29*	.31*	.02	-				
10. Wisconsin^g^	14.09	8.62	.23*	.29*	.32*	.16	.10	.09	.29*	.10	.44*	-			
11. Intra Extra Shift ^h^	8.42	8.92	.14	.17	.16	.12	.19	.13	.17	-.02	.25*	.27*	-		
12. Trails ^i^	479.8	568.7	.12	.14	.15	.13	.03	.04	.18	.02	.10	.15	.13	-	
13. Switching ^j^	-0.05	0.06	.00	-.06	-.08	.12	-.04	-.05	.02	-.01	.01	.00	.01	.09	-

Spearman correlations. N = 344. M is used to represent mean values. SD is used to represent standard deviation

*Correlation is significant at the Bonferroni-adjusted α ≤ .6.00E-05 level (two-tailed)

^a^Alternate Uses: Fluency indicate participants’ number of valid (i.e., unusual) ideas generated in 3 minutes

^b^Alternate Uses: Originality (Max 3) indicate participants’ top 3 most creative responses averaged across the different Alternate Uses Task items

^c^Alternate Uses: Originality (Snapshot) indicate participants’ overall creative responses averaged across the different Alternate Uses Task items

^d^Working Memory is measured by the number of total errors made at all stages in the CANTAB Spatial Working Memory task

^e^Stroop is measured by the Switch-cost (accuracy between congruent versus incongruent trials)

^f^Ravens is measured by total number of correct responses in the Raven’s Standard Progressive Matrices task

^g^Wisconsin indicates performance indicator in the Wisconsin Card Sorting Task

^h^Intra-Extra Shift indicates performance indicator in the CANTAB Intra- and Extra-Dimensional Shift Task

^i^Trails indicates performance indicator in the Trails-Making Task

^j^Switching indicates performance indicator in the Task Set Switching task

As a control analysis, we performed Fisher’s *z*-transformation tests to test whether the correlations between the variables with significant correlations were not statistically different from each other in between the individual and grouped data collection settings. Correlations in the two data collection settings were not statistically different for any of the investigated variables (see Table S1 in Supplementary Information). Given this finding, we ran all subsequent analysis within two settings altogether.

We analysed how the students’ divergent thinking abilities (i.e., fluency and originality) were related to their age, parents’ latest educational level, and their performance in cognitive flexibility (i.e., Wisconsin, Intra Extra Shift, Trails and Switching), working memory, inhibitory control (Stroop), and abstract reasoning (Ravens) tasks. After Bonferroni correction for multiple comparisons, we found that fluency in divergent thinking was significantly correlated with Wisconsin (*r* = .23, *p* = 2.28E-05). Regarding originality in divergent thinking coded either by the Max 3 Scoring or the Snapshot methods, we found it was significantly correlated with Age (r_Max3_ = 0.34, *p* = 3.19E-10; r_Snapshot_= 0.32, *p* = 1.27E-09); Mother’s Education (r_Max3_ = 0.27, *p* = 4.67E-07; r_Snapshot_= 0.30, *p* = 5.53E-07), Working Memory (r_Max3_ = 0.27, *p* = 3.38E-07; r_Snapshot_= 0.28, *p* = 2.12E-07), Ravens (r_Max3_ = 0.29, *p* = 7.19E-08; r_Snapshot_= 0.29, *p* = 1.38E-07), and Wisconsin (r_Max3_ = 0.29, *p* = 2.22E-07; r_Snapshot_= 0.32, *p* = 7.29E-09). The scatterplots for these main findings are highlighted in Supplementary Information (Figures S2 – S4). 

### Hierarchical regression – linear mixed-effect models

To further examine the effect of students’ cognitive performance on their divergent thinking abilities (i.e., fluency and originality) while accounting for the different assessment modes (i.e., Setting), linear mixed-effects hierarchical regression models with random intercepts for Setting (i.e., grouped vs. individualised) were carried out (Tables 3 and 4). We ran parallel analysis including originality in the Alternate Uses coded using the Max 3 and Snapshot scoring methods as dependent variables. As the last scoring method (i.e., Snapshot) yielded the highest predictive values, we are reporting those in the main text (Table 4). Nevertheless, we report the results using the originality scores coded according to the Max3 method in the Supplementary Information (Table S2).

**Table 3:** Hierarchical Regression Results with Linear Mixed-Effects Models with Random Intercept for Setting.Dependent variable: Alternate Uses (Fluency)

Variable	B	p	95% CI for B		SE B	β	R²	ΔR²
			LL	UL				
Step 1							.03	
Constant	.03	.80	-.19	.25	.11	.01		
Age	.14*	.03	.01	.27	.07	.14*		
Mother's Education	.12	.12	-.03	.27	.08	.12		
Father's Education	-.05	.56	-.20	.11	.08	-.05		
Step 2							.08	.05
Constant	.08	.23	-.05	.22	.07	.00		
Age	.16**	.01	.04	.27	.06	.15**		
Mother's Education	.11	.15	-.04	.26	.08	.11		
Father's Education	-.08	.31	-.23	.07	.08	-.08		
Working Memory	.09	.14	-.03	.21	.06	.09		
Stroop	-.11*	.04	-.22	-.00	.06	-.11*		
Ravens	.09	.09	-.01	.20	.05	.10		
Step 3							.11	.03
Constant	.04	.55	-.09	.18	.07	.00		
Age	.14*	.01	.03	.26	.06	.14*		
Mother's Education	.10	.18	-.05	.25	.08	.10		
Father's Education	-.06	.40	-.22	.09	.08	-.06		
Working Memory	.05	.37	-.06	.17	.06	.05		
Stroop	-.12*	.03	-.23	-.01	.06	-.12*		
Ravens	.03	.64	-.09	.14	.06	.03		
Wisconsin	.16*	.01	.04	.29	.06	.16*		
Intra Extra Shift	.00	.97	-.12	.12	.06	.00		
Trails	.08	.16	-.03	.19	.06	.08		
Switching	-.06	.31	-.17	.05	.06	-.06		

*B* fixed-effect coefficient, *SE* Standard error, *CI* 95% Confidence interval, *β* standardized fixed effect computed per imputed dataset

R² values are Nakagawa marginal R². ΔR² is shown as the difference in mean marginal R² across steps. Multiple imputed datasets = 40. Sample size (after imputation): 344. *p ≤ .05. **p ≤ .01. ***p ≤ .001

^a^Working Memory: CANTAB Spatial Working Memory task

^b^Ravens: Raven’s Standard Progressive Matrices task

^c^Wisconsin: Wisconsin Card Sorting Task

^d^Intra-Extra Shift: CANTAB Intra- and Extra-Dimensional Shift Task

^e^Trails: Trails-Making Task A and B

^f^Switching: Task Set Switching task

**Table 4:** Hierarchical Regression Results with Linear Mixed-Effects Models with Random Intercept for Setting.Dependent variable: Alternate Uses (Originality, Snapshot)

Variable	B	p	95% CI for B		SE B	β	R²	ΔR²
			LL	UL				
Step 1							.11	
Constant	.02	.94	-.37	.40	.20	.02		
Age	.20**	.00	.08	.32	.06	.20**		
Mother's Education	.18**	.01	.04	.32	.07	.18**		
Father's Education	.06	.37	-.07	.20	.07	.06		
Step 2							.18	.07
Constant	.08	.53	-.18	.35	.13	.01		
Age	.20***	.00	.09	.32	.06	.21***		
Mother's Education	.17*	.01	.04	.30	.07	.17*		
Father's Education	.02	.72	-.11	.16	.07	.02		
Working Memory	.12*	.02	.02	.23	.05	.12*		
Stroop	-.07	.19	-.17	.03	.05	-.07		
Ravens	.11*	.02	.02	.21	.05	.13*		
Step 3							.23	.05
Constant	.04	.79	-.23	.30	.14	.01		
Age	.20***	.00	.08	.31	.06	.20***		
Mother's Education	.16*	.02	.03	.29	.07	.16*		
Father's Education	.04	.58	-.09	.17	.07	.04		
Working Memory	.09	.11	-.02	.19	.05	.09		
Stroop	-.08	.13	-.18	.02	.05	-.08		
Ravens	0.04	.44	-.06	.14	.05	.05		
Wisconsin	.19**	.00	.08	.30	.06	.19**		
Intra Extra Shift	-.00	.98	-.11	.11	.06	-.00		
Trails	.08	.12	-.02	.18	.05	.08		
Switching	-.15**	.00	-.24	-.05	.05	-.15**		

*B* fixed-effect coefficient, *SE* Standard error, *CI* 95% confidence interval, *β* standardized fixed effect computed per imputed dataset

R² values are Nakagawa marginal R². ΔR² is shown as the difference in mean marginal R² across steps. Multiple imputed datasets = 40. Sample size (after imputation): 344. *p ≤ .05. **p ≤ .01. ***p ≤ .001

^a^Working Memory: CANTAB Spatial Working Memory task

^b^Ravens: Raven’s Standard Progressive Matrices task

^c^Wisconsin: Wisconsin Card Sorting Task

^d^Intra-Extra Shift: CANTAB Intra- and Extra-Dimensional Shift Task

^e^Trails: Trails-Making Task A and B

^f^Switching: Task Set Switching task

Prior to building the linear mixed-effects models, we calculated intraclass correlation coefficient values to assess the extent of clustering in the two Settings [96], in relation to the different dependent variables. When calculating the intraclass correlation coefficient (ICC) for fluency, it yielded a low, but non-negligible coefficient value (ICC = 0.07). This indicates that 7% of the variance in the Alternate Uses: Fluency was attributable to differences between the assessment modes. For the originality metrics in the Alternate Uses (i.e., Max-3 and Snapshot), both yielded moderate intraclass correlation indexes (i.e., ICC = 0.26 and 0.24; respectively). This means that approximately one quarter of the variance in these outcomes was attributable to clustering between the two assessment modes, substantiating the use of mixed-effects models with random intercepts for Setting, particularly for the Alternate Uses Originality-based outcomes. Although the intraclass correlation value using the fluency variable is considered low [97], we include the mixed-effects models for this metric as well to ensure homogenous reporting standards across the dependent variables.

For the hierarchical regressions adopting linear mixed-effects models with random intercepts, when considering fluency in divergent thinking as the dependent variable, participants’ age and their family demographic variables (i.e., Mother’s Education and Father’s Education) in the first model, only Age showed statistically significant associations with the dependent variable (*β* = 0.14, *p* = .03). Step 1 accounted for 3% of the variance in the dependent variable. When Working Memory, Stroop and Ravens were introduced in Step 2 as predictors, Stroop (*β* = − 0.11, *p* = .04) significantly influenced fluency in divergent thinking, while Age (*β* = 0.16, *p* = .01) continued to hold a statistically significant influence on the dependent variable. They explained an additional 5% of the variance in the students’ fluency in divergent thinking, providing a significant improvement in its explanatory power when compared to Step 2 (D2 pooled test, *F* (3, 3703.0) = 3.545, *p* = .01). In Step 3, we added Wisconsin, Intra-Extra Shift, Trails, and Switching as predictors. In this last step, Age (*β* = 0.14, *p*= .01), Stroop (*β* = − 0.12, *p*= .03) and Wisconsin (*β* = 0.16, *p*= .01) were significant predictors of fluency in divergent thinking. In Step 3, the model significantly explained an additional 3% of the variance in the dependent variable (D2 pooled test, *F* (4, 5.640.3) = 2.86, *p* = .02).

When considering originality in divergent thinking measured by the Snapshot method as the dependent variable, participants’ age and their family demographic variables (i.e., Mother’s Education and Father’s education) in the Step 1, Age (*β* = 0.20, *p* = .001) and Mother’s Education (*β* = 0.18, *p* = .01) had a significant effect. Step 1 accounted for 11% of the variance in the dependent variable. When Working Memory, Stroop and Ravens were introduced in Step 2 as predictors, Working Memory (*β* = 0.13, *p* = .02) and Ravens (*β* = 0.11, *p* = .02) significantly influenced originality in divergent thinking, while Age (*β* = 0.20, *p* = .01) and Mother’s Education (*β* = 0.16, *p* = .01) continued to hold a statistically significant influence on the dependent variable. The inclusion of variables in Step 2 explained additional 7% of the variance in the students’ overall originality in divergent thinking, with a significant improvement in its explanatory power when compared to Step 1 (D2 pooled test, *F* (3, 4070.7) = 5.78, *p* = .001). In Step 3, we added Wisconsin, Intra-Extra Shift, Trails, and Switching as predictors. In this last step, Wisconsin (*β* = 0.19, *p* = .001) and Switching (*β* = − 0.15, *p* = .003) were significant predictors of overall originality in divergent thinking. Age (*β* = 0.20, *p* = 9.00E-04), and Mother’s Education (*β* = 0.16, *p*= .02) continued to provide a statistically significant influence on the dependent variable. In Step 3, the model significantly explained an additional 5% of the variance in the dependent variable (D2 pooled test, *F* (4, 1857.5) = 5.84, *p* < .001).

When we tested the multicollinearity (i.e., Variance Inflation Factor; VIF) for the dependent variables included in all final regression models, we found the following VIF values: (1) Age = 1.11, (2) Mother’s Education = 1.71, (3) Father’s Education = 1.78, 3) Working Memory = 1.21, (4) Stroop = 1.03, (5) Ravens = 1.47, (6) Wisconsin = 1.38, (7) Intra-Extra Shift = 1.23, (8) Trails = 1.08, and 8) Switching = 1.02. All predictors showed VIF values well below the recommended cutoffs (i.e., < 5; [95]), suggesting that multicollinearity did not bias our regression estimates.

In summary, when considering fluency in divergent thinking as the dependent variable (Table 3), in addition to Age; inhibitory control (Stroop) and set-shifting (Wisconsin) abilities show unique contributions in the final model: better Stroop performance (i.e., higher inhibitory control abilities) relates to lower fluency in divergent thinking, whereas better Wisconsin performance (i.e., higher set-shifting) relates to higher fluency. For originality in divergent thinking (Table 4), in addition to Age and Mother’s Education, the cognitive variables contributing most to the variance of the dependent variable are Wisconsin and Switching. Wisconsin had the highest contribution to originality in divergent thinking when compared to the other variables, with higher performance (i.e., less perseverative behaviours) leading to higher originality scores in the Alternate Uses task. Task-switching (i.e., Switching) abilities have a negative influence on originality in divergent thinking, with higher task-switching performance (i.e., better accuracy in switching between two tasks) leading to lower originality. Finally, it is important to note that these results are significant while accounting for models testing linear mixed effects with random intercepts, clustering across the different Setting conditions (i.e., grouped and individualized). This means that the influence of the independent variables, especially the ones referring to set-shifting and task-switching in cognitive flexibility, on the dependent variables (i.e., divergent thinking indicators) remained invariant across the different assessment modes. These findings highlight how the use of different tasks could allow us to tease apart distinct cognitive flexibility subprocesses whose unique contributions emerge even when overlapping executive function, and demographic influences, and possible differences of data collection settings are controlled for.

## Discussion

Our findings provide novel evidence that cognitive flexibility significantly contributes to divergent thinking in adolescents, with distinct roles emerging within its subcomponents. Specifically, set-shifting, entailed here by performance in the Wisconsin Card Sorting, is a positive predictor of fluency (i.e., the number of ideas generated), and originality (i.e., how creative participants were) in a divergent thinking task (i.e., the Alternate Uses). Meanwhile, task-switching, entailed here as performance in the Task-Set Switching, was a negative predictor of originality in divergent thinking. This dissociation suggests that distinct facets of cognitive flexibility underpin divergent thinking through separate mechanisms, with the first (i.e., set-shifting) acting as a facilitator of divergent thinking performance while the second (i.e., task-switching) potentially hindering the quality of the different generated ideas.

These results remained invariant across the different assessment modes, which means that the influences of the independent variables on the divergent thinking metrics are not different across grouped and individualized settings. Such nuanced results underscore the multifaceted nature of cognitive flexibility in creativity, highlighting that each ability may support distinct dimensions of creative performance in youth. This extends prior research emphasizing the roles of working memory [75] and inhibitory control [76] in divergent thinking.

### Cognitive flexibility as a complex and multifaceted construct

A key methodological advance of our study lies in the use of a comprehensive range of cognitive flexibility tasks. As noted in the Introduction section, across the literature there are various experimental paradigms used to index cognitive flexibility [23, 34, 35]. However, because there is this growing consensus on conceptualizing cognitive flexibility as a multifaceted construct [98, 99], we amplified the understanding about the cognitive processes underpinning divergent thinking during adolescence by using a broad set of widely recognized cognitive flexibility tasks in a single sample. This approach is justified by indications that empirical evidence linking cognitive flexibility to divergent thinking, especially during adolescence, remains limited and inconsistent [7]. Besides, a closer examination of prior research tackling the contribution of cognitive flexibility to divergent thinking during adolescence years reveals inconsistencies in task selection across different studies.

For instance, Krumm et al. [48] found that, after controlling for intelligence and inhibition, cognitive flexibility contributed to creative performance in children (i.e., 8- to -13-year-olds). However, while one of their tasks aligned with set-shifting paradigms (i.e., Wisconsin Card Sorting), others (i.e., semantic verbal/figural fluency tasks) could be rather interpreted as indicators of associative memory [75] or creative idea generation [100] processes. Similarly, Arán Filippetti & Krumm [57] used similar tasks as cognitive flexibility indicators in children (8- to 12-year-olds) and found that only tasks related to spontaneous flexibility (i.e., Verbal Fluency and the Five-Point Test) contributed to creativity. As highlighted by Palmiero et al. [7], this overlap between divergent thinking and cognitive flexibility indicators may complicate the interpretation of findings. Meanwhile, Peláez-Alfonso [58] reported no significant relationship between performance in a task-switching task (e.g., number–letter and plus–minus) and divergent thinking among adolescents, which aligns with some of the findings from adult populations [49, 94].

Differences between their results and ours may stem from neural and behavioural responses tied to specific cognitive flexibility subcomponents [34, 99, 101, 102]. For example, previous findings indicate that simple task-switching tasks might focus on attention-shifting but might not engage broad cognitive flexibility competencies [103, 104]. Meanwhile, a meta-analytic study identified that set-shifting tasks (e.g., Wisconsin) may require a wider range of brain structures, such as higher-order prefrontal and cortico-striatal systems in addition to frontoparietal attention networks, supporting rule discovery in cognitive flexibility [68]. Our findings support the evidence that different cognitive flexibility abilities (i.e., task switching and set-shifting), together with additional cognitive functions, likely mobilize distinct cognitive processes, which, in turn, may have competing and facilitatory influences on divergent thinking in adolescence. Had we included only a single cognitive flexibility task or focused in only one cluster of abilities (e.g., task-switching), such nuanced effects might have been masked. Our comprehensive approach sheds new light on the nuanced role of cognitive flexibility in adolescent creativity and highlights the importance of task selection in accurately capturing its contribution.

### Cognitive flexibility abilities within the cognitive shifting subcomponent influence divergent thinking, together with additional cognitive processes and socio-demographical factors

A closer analysis of our correlations indicates that cognitive flexibility and other general cognitive resources were differentially associated with divergent thinking outcomes. Specifically, older adolescents and those with superior set-shifting performance in the Wisconsin task generated significantly more ideas, whereas no notable associations with other cognitive flexibility and executive functions abilities (e.g., task-switching, working memory, inhibition, and abstract reasoning) were significant after Bonferroni correction for multiple comparisons. Meanwhile, originality performance (entailed by both the Max 3 and Snapshot metrics) were significantly positively correlated with age, maternal education, working memory capacity, abstract reasoning (i.e., Ravens), and set-shifting performance. This pattern suggests that the ability to flexibly shift mental sets in a self-guided way (i.e., by having participants to figure out a new rule by themselves) facilitates both the quantity and quality of ideas, aligning with the view that approaching problems from multiple angles enhances creativity [7, 23, 53–55].

Moreover, the links between originality and higher working memory capacity and abstract reasoning indicate that greater cognitive resources (e.g., the capability to maintain and manipulate information and to reason abstractly) support the production of more novel ideas, and aligns with previous findings [7, 105, 106]. Finally, the positive associations of age and maternal education, which is often used as proxy of socioeconomic status [107], with both fluency and originality align with previous literature emphasizing the importance of developmental and environmental influences on creativity [108].

Subsequently, in our linear mixed-effects hierarchical regression models, task-switching and set-shifting measures showed differential links to divergent thinking outcomes (i.e., fluency and originality). Notably, after accounting for demographics and additional cognitive variables (namely, age, parental education, working memory, inhibitory control and abstract reasoning), performance on the Wisconsin task predicted higher fluency and originality scores, whereas accuracy in Task-Set Switching, which is a cue-guided task was associated with lower originality.

These novel findings suggest that the capacity for a self-guided set-shifting, as captured by Wisconsin, may foster broad associative exploration of the semantic space (i.e., the organization of concepts in memory according to semantic relatedness [109]), and novel idea generation in the Alternate Uses. More specifically, successful performance of the Wisconsin involves rule discovery [68] overcoming perseveration (i.e., not sticking to an old rule) and being open to try new hypotheses [110]. In this paradigm, participants do not have access to the sorting rule, and they must figure it out through continuous monitoring of the outcome after some rounds of trial-and-error attempts [68]. This type of cognitive flexibility ability may be beneficial to divergent thinking – and more broadly, to creativity – as it may allow individuals to break out of habitual thinking patterns and consider alternative categories or interpretations of a problem. This can help them to think about a greater variety of ideas (i.e., higher fluency) and to find more novel ideas (i.e., increase originality). In summary, set-shifting ability as captured by the Wisconsin task may provide the mental capacity to explore ideas broadly, preventing stagnation in an inefficient line of thought and thereby fostering fluency and originality in divergent thinking.

Meanwhile, adolescents’ performance on our task set-switching task, which required participants to switch their response every time they identified a certain cue, may reflect a rapid, and temporally constrained cognitive flexibility ability that may potentially hinder originality of diverse ideas. In this regard, Wu and Koutstaal [111] show parallel results in an adult sample, demonstrating that when participants were required to switch regularly between ideas in the Alternate Uses, they had lower performance in originality, compared to when they were instructed to stay longer in one task item or to shift whenever they wish. Altogether, the findings from this study suggest that being too responsive to environmentally guided switching cues might come at the expense of deeper exploration of a given idea. Originality in creative thinking often requires a certain degree of persistence or incubation by sticking with a thought long enough to go through into less obvious aspects of a problem or to combine ideas in novel ways [55, 111]. If someone’s tendency is to “shift gear” very readily, either due to a tendency toward frequent switching or a short attention span, they might produce many ideas quickly, but those ideas will likely be superficial or conventional. This will, therefore, inhibit the elaboration needed for true originality.

Such opposing contributions from cognitive flexibility’s set-shifting and task-switching abilities echo the dual-pathway framework of creativity, wherein creativity can stem either from flexible switching between diverse concepts or from persistent, focused processing of a few ideas in depth [55]. In line with this view, our findings indicate that divergent thinking may benefit more from a self-guided type of set-shifting ability as exhibited in the Wisconsin task, where participants have to figure out by themselves when to shift according to a new correct rule, compared to when they simply have to switch their responses whenever it is instructed [68]. This underscores the importance of an optimal interplay between different cognitive flexibility abilities for optimal creative output.

Lastly, we would like to highlight the contribution of inhibitory control to the fluency aspect in divergent thinking. Specifically, we found that, after accounting for other cognitive measures, better performance in Stroop has a significant negative effect on the number of generated ideas in the Alternate Uses Task. It is important to point out that the association between inhibitory control and creativity is complex, with relationships between the two variables depending on the type of task implemented and the developmental stage of the participants [7]. Some studies in adults (e.g. [112–114]), have found that inhibitory control can promote fluency by, for example, inhibiting obvious or common responses to prevent fixation on the most typical ideas, whereas others could not identify significant influences of inhibitory control on divergent thinking or found that low inhibition abilities could lead to higher divergent thinking performance (for more details, see Palmiero et al. [7]). In children and adolescents, authors using specific tasks for inhibitory control (e.g., Stroop, Flanker or Go/No-Go) to investigate its influence on divergent thinking did not find significant results [115–117], although one study identified a positive association [49].

Nevertheless, as Stroop is a type of inhibitory control task that emphasizes set maintenance by resisting to the interference of a dominant stimulus [118]; in the present study, we hypothesize that higher performance in this task may indicate high levels of inhibitory control abilities that potentially prevent diversification of creative outputs during adolescence. This aligns with evidence, in adult participants, showing that lower inhibitory capacity leads to higher levels of idea generation in divergent thinking tasks [119, 120]. In adolescents, inhibitory control may stifle the free-flow process of generating unusual ideas by possibly suppressing out-of-the-box associations that could have rather resulted in creative solutions. This, in turn, may impact fluency in divergent thinking by constraining the associative breadth during idea generation. Albeit interesting, these findings warrant further investigation by future studies.

Overall, the current work highlights the nuanced relationship between cognitive flexibility and divergent thinking during adolescence. As we discussed earlier in the Introduction section, others have identified cognitive flexibility as including a set of different abilities [34, 35], with tasks commonly used in the literature differing in the extent to which they emphasize self-guided set-shifting, or externally cued task-switching [68]. Moreover, evidence suggests that these tasks are likely recruiting overlapping brain networks involved in working memory, inhibitory control, and broader cognitive control processes [68]. In adolescence, when cognitive systems are still developing [38], the contribution of these additional cognitive processes is likely important to support cognitive flexibility and divergent thinking. For example, de Chantal et al. [121] found that metrics of working memory and cognitive flexibility altogether had a significant contribution to divergent thinking in children aged from 10 to 12 years-old. In our study, although cognitive flexibility can be viewed as operating under the broader umbrella of cognitive control processes, we could observe distinct subcomponents (i.e., set-shifting and task- switching) differentially supporting idea generation and idea selection in divergent thinking. Clarifying these distinctions may help reconcile mixed findings in the literature [7, 49, 56–58] and underscores the importance of considering task characteristics when interpreting links between cognitive flexibility and creativity processes in developmental samples.

### Limitations and future directions

Our results provide an important step in understanding the cognitive abilities underpinning creativity. However, there are some limitations that could be addressed in future research. Due to time restrictions and to prevent participant fatigue, we used a limited set of tasks comprising cognitive abilities other than cognitive flexibility (i.e., one task for working memory, one task for inhibitory control and one task for abstract reasoning), and just one task entailing creative divergent thinking (i.e., Alternate Uses Task). In future studies, we plan to implement tasks comprising other aspects of creative thinking, such as the Remote Associates Task [122] for convergent thinking or the Multi Trial Creative Ideation (MTCI) task [123] for figural creativity.

Moreover, expanding the range of participant ages is important for understanding the role of cognitive flexibility across development. Past research has found developmental differences in cognitive flexibility, when comparing younger and older adolescents [124], with high levels of dynamic changes happening within the younger group. Also, earlier findings indicate developmental differences in creativity tasks from early childhood (around 6 years old) to late adolescence (around 17 years old), with evidence of a creativity performance decrease typically occurring between ages 12 and 13 [51]. Therefore, we focused on an age range in early adolescence to capture a developmental window in which cognitive abilities are undergoing dynamic and malleable changes, with critical implications for the development of creative abilities. Future studies may benefit from investigating whether within a broader age range the relationship between cognitive flexibility, and divergent thinking along adolescence would still be kept uniform.

Another limitation of the present study concerns about the variability in the testing context during individualized sessions. While participants tested in a 1:2 ratio were assessed in separate rooms and did not interact, recent work suggests that the mere presence of others, even without direct interaction can modulate how children and adolescents engage cognitive functions through attentional and motivational processes [125, 126]. Although such effects within the individualized assessment mode were not the focus of the present study, and despite the fact we did not find differences in results between assessment modes/settings, future research may systematically manipulate and control for social presence to better disentangle its role in cognitive and creative performance during adolescence.

Finally, although some tasks in our testing battery, particularly the one assessing task-switching, showed relatively low internal reliability, this limitation should be interpreted within the broader context of cognitive task design. As Hedge et al. [127] states, many robust experimental tasks are constructed to minimise between-subject variability so that they produce strong, replicable group-level effects; however, this same feature can reduce their sensitivity to individual differences. Despite this constraint, such tasks remain valuable because they reliably capture well-established cognitive phenomena and allow researchers to probe theoretically meaningful processes. In our study, even measures with low to modest reliability yielded useful performance indicators that, when interpreted alongside converging evidence from other executive function tasks, supported a more nuanced picture of the cognitive mechanisms underlying divergent thinking.

## Implications and conclusions

In conclusion, to our knowledge, ours are the first findings to address the unique contribution of different abilities (i.e., set-shifting and task-switching) within the cognitive flexibility construct to divergent thinking in a large sample of adolescents. Adolescence is a pivotal period characterized by biological, cognitive, and social changes, during which individuals make critical life decisions regarding relationships and career paths. In this context, cognitive flexibility and divergent thinking are crucial abilities, especially in the school environment, where social interactions and goal-oriented behaviour demand adaptability and innovation [128]. Success in school often hinges on flexible and creative thinking, as argued by Diamond [129]. Cognitive flexibility is not only critical for mastering specific academic subjects but also for navigating transitions between classroom activities, enabling students to adjust their focus and responses to meet new demands [130]. Furthermore, flexible thinking has been shown to aid in situations requiring rapid adaptation and the generation of novel solutions [131].

Given the importance of fostering creativity and adaptability during the school years, educational interventions targeting cognitive flexibility may have profound benefits. For example, according to the present findings, interventions targeting cognitive flexibility training may need careful consideration of the specific paradigms employed, as we demonstrated that performance in task-set switching and set-shifting tasks can have opposing effects to divergent thinking in adolescents. This indicates that training protocols aiming to foster creativity should move beyond pure switching-based paradigms and instead incorporate processes such as flexible rule inference, and exploratory strategy use that are more closely aligned with divergent thinking. As Lee et al. [132] emphasize, cultivating cognitive flexibility early in development (e.g., by employing educational interventional programs) can serve as a foundation for innovation and divergent thinking, equipping students to thrive academically and in real-world scenarios.

## Supplementary Information

Supplementary Material 1.

## Data Availability

The analysis scripts are openly available in OSF (https://osf.io/xf78p/files/osfstorage). The dataset analysed in the current study is available at the DR-NTU Data Repository: https://doi.org/10.21979/N9/NFZ89Q .
