# Supplementary material for: Set-shifting and task-switching make differential contributions to divergent thinking in adolescence
Source: BMC Psychol. 2026 Apr 28;14:733. doi: 10.1186/s40359-026-04584-5 (PMC13185229; doi:10.1186/s40359-026-04584-5)
Supplement: Supplementary file 1 — Supplementary Material 1. [file 40359_2026_4584_MOESM1_ESM.docx]

**Supplementary Information**

**Results**

**Table S1**

*Fisher’s Z-tests Comparisons of Correlations in between Individualised (N=154) and Grouped (N=190) Assessments*

| ***Comparing correlations between the two settings*** | | | | | | | | | |
| --- | --- | --- | --- | --- | --- | --- | --- | --- | --- |
|  | **r1 (IA)** | **r2 (GA)** | **95% CI - IA** | | **95% CI - GA** | | **z score** | **p-value** | **Cohen's q** |
|  | **Lower** | **Upper** | **Lower** | **Upper** |  | | |  |  |
| Alternate Uses: Fluency vs. Alternate Uses: Originality (Max 3) | .689 | .586 | .591 | .767 | .479 | .676 | 1.601 | .109 | .175 |
| Alternate Uses: Fluency vs. Alternate Uses: Originality (Snapshot) | .600 | .489 | .485 | .695 | .367 | .595 | 1.445 | .149 | .158 |
| Alternate Uses: Fluency vs. Age | .069 | .069 | -.098 | .232 | -.077 | .212 | -.000 | 1.000 | -.000 |
| Alternate Uses: Fluency vs. Mother's Education | .129 | .126 | -.045 | .296 | -.035 | .280 | .032 | .975 | .003 |
| Alternate Uses: Fluency vs. Father's Education | .013 | .065 | -.158 | .184 | -.096 | .222 | -.472 | .637 | -.052 |
| Alternate Uses: Fluency vs. Working Memory | .155 | .080 | -.005 | .307 | -.069 | .226 | .693 | .489 | .076 |
| Alternate Uses: Fluency vs. Stroop | -.090 | -.082 | -.249 | .073 | -.228 | .069 | -.077 | .939 | -.008 |
| Alternate Uses: Fluency vs. Ravens | .073 | .152 | -.088 | .231 | .004 | .294 | -.730 | .465 | -.080 |
| Alternate Uses: Fluency vs. Wisconsin | .137 | .191 | -.024 | .290 | .041 | .331 | -.508 | .611 | -.056 |
| Alternate Uses: Fluency vs. Intra-Extra Shift | .026 | .139 | -.141 | .192 | -.017 | .288 | -1.035 | .301 | -.113 |
| Alternate Uses: Fluency vs. Trails | .210 | .033 | .048 | .361 | -.121 | .184 | 1.648 | .099 | .180 |
| Alternate Uses: Fluency vs. Switching | -.094 | -.009 | -.250 | .067 | -.162 | .143 | -.775 | .438 | -.085 |
| Alternate Uses: Originality (Max 3) vs. Alternate Uses: Originality (Snapshot) | .947 | .921 | .927 | .961 | .894 | .941 | 1.868 | .062 | .204 |
| Alternate Uses: Originality (Max 3) vs. Age | .224 | .041 | .064 | .373 | -.106 | .186 | 1.708 | .088 | .187 |
| Alternate Uses: Originality (Max 3) vs. Mother's Education | .155 | .225 | -.021 | .321 | .065 | .374 | -.666 | .505 | -.073 |
| Alternate Uses: Originality (Max 3) vs. Father's Education | .145 | .129 | -.027 | .308 | -.033 | .283 | .151 | .880 | .017 |
| Alternate Uses: Originality (Max 3) vs. Working Memory | .159 | .151 | -.000 | .310 | .002 | .293 | .073 | .942 | .008 |
| Alternate Uses: Originality (Max 3) vs. Stroop | -.020 | -.059 | -.180 | .142 | -.206 | .090 | .361 | .718 | .040 |
| Alternate Uses: Originality (Max 3) vs. Ravens | .235 | .208 | .078 | .380 | .061 | .346 | .254 | .800 | .028 |
| Alternate Uses: Originality (Max 3) vs. Wisconsin | .180 | .203 | .020 | .331 | .049 | .347 | -.215 | .829 | -.024 |
| Alternate Uses: Originality (Max 3) vs. Intra-Extra Shift | .098 | .095 | -.071 | .262 | -.064 | .250 | .028 | .978 | .003 |
| Alternate Uses: Originality (Max 3) vs. Trails | .160 | .109 | -.001 | .313 | -.044 | .256 | .475 | .635 | .052 |
| Alternate Uses: Originality (Max 3) vs. Switching | -.109 | -.132 | -.264 | .051 | -.279 | .020 | .215 | .830 | .024 |
| Alternate Uses: Originality (Snapshot) vs. Age | .234 | .040 | .073 | .383 | -.106 | .185 | 1.811 | .070 | .198 |
| Alternate Uses: Originality (Snapshot) vs. Mother's Education | .126 | .295 | -.047 | .291 | .143 | .434 | -1.628 | .103 | -.178 |
| Alternate Uses: Originality (Snapshot) vs. Father's Education | .107 | .175 | -.062 | .271 | .016 | .324 | -.627 | .531 | -.069 |
| Alternate Uses: Originality (Snapshot) vs. Working Memory | .169 | .144 | .010 | .320 | -.004 | .287 | .234 | .815 | .026 |
| Alternate Uses: Originality (Snapshot) vs. Stroop | -.032 | -.040 | -.194 | .131 | -.186 | .108 | .070 | .944 | .008 |
| Alternate Uses: Originality (Snapshot) vs. Ravens | .232 | .189 | .075 | .377 | .042 | .328 | .409 | .683 | .045 |
| Alternate Uses: Originality (Snapshot) vs. Wisconsin | .207 | .246 | .049 | .355 | .095 | .385 | -.372 | .710 | -.041 |
| Alternate Uses: Originality (Snapshot) vs. Intra-Extra Shift | .093 | .087 | -.075 | .255 | -.069 | .239 | .051 | .959 | .006 |
| Alternate Uses: Originality (Snapshot) vs. Trails | .130 | .130 | -.031 | .284 | -.024 | .279 | -.008 | .994 | -.001 |
| Alternate Uses: Originality (Snapshot) vs. Switching | -.139 | -.138 | -.292 | .021 | -.285 | .015 | -.005 | .996 | -.001 |
| Age vs. Mother's Education | -.054 | -.022 | -.229 | .124 | -.175 | .132 | -.292 | .770 | -.032 |
| Age vs. Father's Education | -.070 | -.019 | -.242 | .106 | -.170 | .133 | -.469 | .639 | -.051 |
| Age vs. Working Memory | .103 | -.014 | -.065 | .265 | -.157 | .129 | 1.071 | .284 | .117 |
| Age vs. Stroop | .280 | -.037 | .124 | .423 | -.181 | .109 | 2.967 | .003 | .325 |
| Age vs. Ravens | .047 | -.036 | -.118 | .209 | -.183 | .111 | .759 | .448 | .083 |
| Age vs. Wisconsin | .095 | -.051 | -.070 | .255 | -.195 | .095 | 1.338 | .181 | .146 |
| Age vs. Intra-Extra Shift | -.048 | .083 | -.213 | .120 | -.073 | .234 | -1.198 | .231 | -.131 |
| Age vs. Trails | .044 | .164 | -.121 | .207 | .017 | .304 | -1.105 | .269 | -.121 |
| Age vs. Switching | .030 | .111 | -.132 | .190 | -.039 | .257 | -.747 | .455 | -.082 |
| Mother's Education vs. Father's Education | .536 | .605 | .406 | .645 | .491 | .699 | -.929 | .353 | -.102 |
| Mother's Education vs. Working Memory | .152 | .091 | -.016 | .312 | -.068 | .246 | .565 | .572 | .062 |
| Mother's Education vs. Stroop | -.025 | -.085 | -.194 | .145 | -.239 | .073 | .553 | .581 | .060 |
| Mother's Education vs. Ravens | .179 | .129 | .014 | .335 | -.031 | .282 | .473 | .636 | .052 |
| Mother's Education vs. Wisconsin | .023 | .034 | -.151 | .195 | -.125 | .190 | -.100 | .920 | -.011 |
| Mother's Education vs. Intra-Extra Shift | .141 | .136 | -.032 | .306 | -.023 | .289 | .046 | .964 | .005 |
| Mother's Education vs. Trails | .025 | .007 | -.146 | .194 | -.156 | .170 | .160 | .873 | .017 |
| Mother's Education vs. Switching | -.039 | -.070 | -.206 | .132 | -.223 | .087 | .287 | .774 | .031 |
| Father's Education vs. Working Memory | .065 | .089 | -.106 | .233 | -.071 | .244 | -.216 | .829 | -.024 |
| Father's Education vs. Stroop | -.091 | -.074 | -.255 | .077 | -.226 | .083 | -.165 | .869 | -.018 |
| Father's Education vs. Ravens | .253 | .197 | .091 | .403 | .042 | .344 | .538 | .590 | .059 |
| Father's Education vs. Wisconsin | .060 | -.013 | -.107 | .223 | -.167 | .141 | .670 | .503 | .073 |
| Father's Education vs. Intra-Extra Shift | .085 | .048 | -.092 | .257 | -.111 | .205 | .337 | .736 | .037 |
| Father's Education vs. Trails | .057 | -.004 | -.119 | .230 | -.160 | .152 | .559 | .576 | .061 |
| Father's Education vs. Switching | -.015 | -.112 | -.184 | .154 | -.266 | .049 | .886 | .376 | .097 |
| Working Memory vs. Stroop | .120 | .153 | -.042 | .276 | .006 | .293 | -.303 | .762 | -.033 |
| Working Memory vs. Ravens | .273 | .186 | .119 | .414 | .038 | .325 | .845 | .398 | .092 |
| Working Memory vs. Wisconsin | .142 | .255 | -.019 | .296 | .110 | .389 | -1.074 | .283 | -.117 |
| Working Memory vs. Intra-Extra Shift | .090 | .143 | -.074 | .250 | -.014 | .294 | -.493 | .622 | -.054 |
| Working Memory vs. Trails | .280 | .085 | .122 | .423 | -.065 | .231 | 1.848 | .065 | .202 |
| Working Memory vs. Switching | -.004 | -.043 | -.164 | .158 | -.189 | .104 | .364 | .716 | .040 |
| Stroop vs. Ravens | .062 | .008 | -.101 | .220 | -.141 | .156 | .492 | .623 | .054 |
| Stroop vs. Wisconsin | .167 | .081 | .006 | .319 | -.067 | .226 | .797 | .425 | .087 |
| Stroop vs. Intra-Extra Shift | .008 | -.010 | -.159 | .175 | -.168 | .148 | .168 | .866 | .018 |
| Stroop vs. Trails | .089 | -.023 | -.075 | .248 | -.177 | .133 | 1.020 | .308 | .112 |
| Stroop vs. Switching | .023 | -.038 | -.139 | .183 | -.187 | .112 | .558 | .577 | .061 |
| Ravens vs. Wisconsin | .304 | .429 | .152 | .443 | .301 | .542 | -1.315 | .188 | -.144 |
| Ravens vs. Intra-Extra Shift | .196 | .220 | .035 | .347 | .061 | .368 | -.229 | .819 | -.025 |
| Ravens vs. Trails | .169 | .017 | .008 | .322 | -.133 | .166 | 1.401 | .161 | .153 |
| Ravens vs. Switching | -.049 | -.008 | -.209 | .113 | -.155 | .139 | -.376 | .707 | -.041 |
| Wisconsin vs. Intra-Extra Shift | .220 | .234 | .056 | .372 | .077 | .380 | -.141 | .888 | -.015 |
| Wisconsin vs. Trails | .196 | .076 | .035 | .346 | -.075 | .223 | 1.117 | .264 | .122 |
| Wisconsin vs. Switching | -.003 | -.039 | -.163 | .158 | -.187 | .111 | .333 | .739 | .036 |
| Intra-Extra Shift vs. Trails | .128 | .111 | -.043 | .292 | -.059 | .274 | .163 | .871 | .018 |
| Intra-Extra Shift vs. Switching | .046 | -.056 | -.124 | .213 | -.212 | .104 | .931 | .352 | .102 |
| Trails vs. Switching | .176 | .022 | .016 | .328 | -.135 | .178 | 1.430 | .153 | .156 |

*Note*. r1 (Individual) = r value for the correlation in between two given variables in the individualised assessment. r2 (Grouped) = r value for the correlation in between two given variables in the grouped assessment. CI = confidence interval. z statistics = Fisher’s z-transformation score. Cohen q = Effect size measure for differences of independent correlations. According to Cohen (1988), q = |.10|, |.30| and |.50| are considered small, moderate, and large differences, respectively. Missing data was imputed using the multiple imputation (MI) method. All correlation values are Spearman’s rho values. Correlation is significant at the Bonferroni-adjusted α=.001 level (two-tailed).

Alternate Uses: Fluency = participants’ number of valid (i.e., unusual) ideas generated in 3 minutes.

Alternate Uses: Originality (Max 3) = participants’ top 3 most creative responses averaged across the different Alternate Uses Task items.

Alternate Uses: Originality (Snapshot) = participants’ overall creative responses averaged across the different Alternate Uses Task items.

Working Memory = number of total errors made at all stages in the CANTAB Spatial Working Memory task.

Stroop = Switch-cost (accuracy between congruent versus incongruent trials).

Ravens = total number of correct responses in the Raven’s Standard Progressive Matrices task.

Wisconsin = performance indicator in the Wisconsin Card Sorting Task.

Intra-Extra Shift = performance indicator in the CANTAB Intra- and Extra-Dimensional Shift Task.

Trails = performance indicator in the Trails Making Task.

Switching = performance indicator in the Task Set Switching task.

**Figure S1**

**
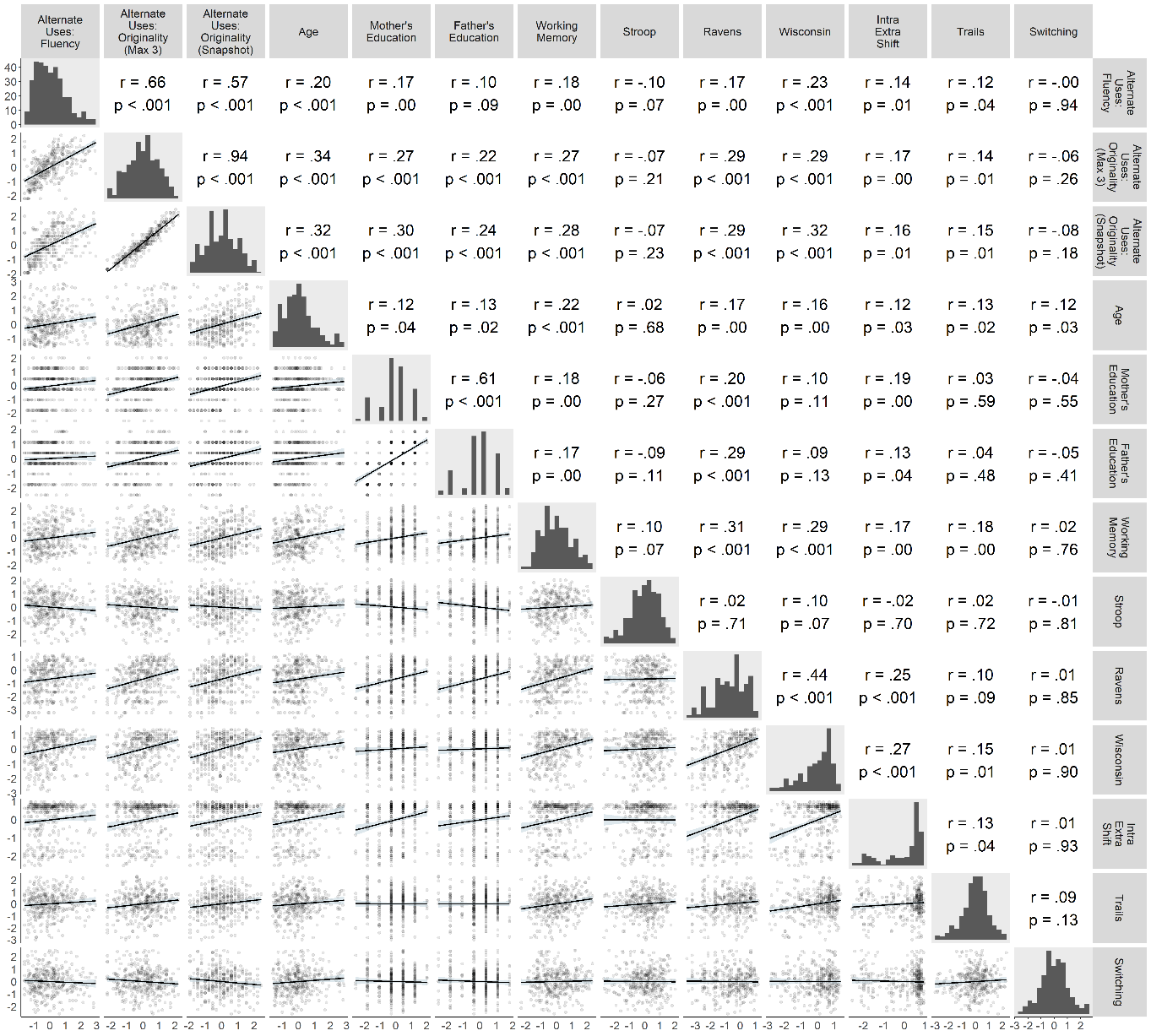
**

*Note*. Correlation coefficients (i.e., exact *r* and *p* values) between the Alternate Uses Task Fluency and Originality (i.e., Max 3 and Snapshot) scores, age, parental (i.e., mother’s and father’s) education, working memory, inhibitory control, Raven’s Standard Progressive Matrices (SPM) and cognitive flexibility tasks performance. Multiple imputed datasets: 40. Sample size (after multiple imputation): 344.

*Alternate Uses: Fluency*: number of valid (i.e., unusual) ideas generated in 3 minutes during the Alternate Uses Task. *Alternate Uses: Originality (Max 3):* indicate participants’ top 3 most creative responses averaged across the different Alternate Uses Task items*.* *Alternate Uses: Originality (Snapshot)*: overall creative responses averaged across the different Alternate Uses Task items. *Working Memory:* CANTAB Spatial Working Memory *task.* *Stroop:* Stroop task. *Ravens:* Raven’s Standard Progressive Matrices task*.* *Wisconsin*: Wisconsin Card Sorting Task. *Intra-Extra Shift*: CANTAB Intra- and Extra-Dimensional Shift Task. *Trails*: Trails Making Task. *Switching:* Task Set Switching task.

Figure S2


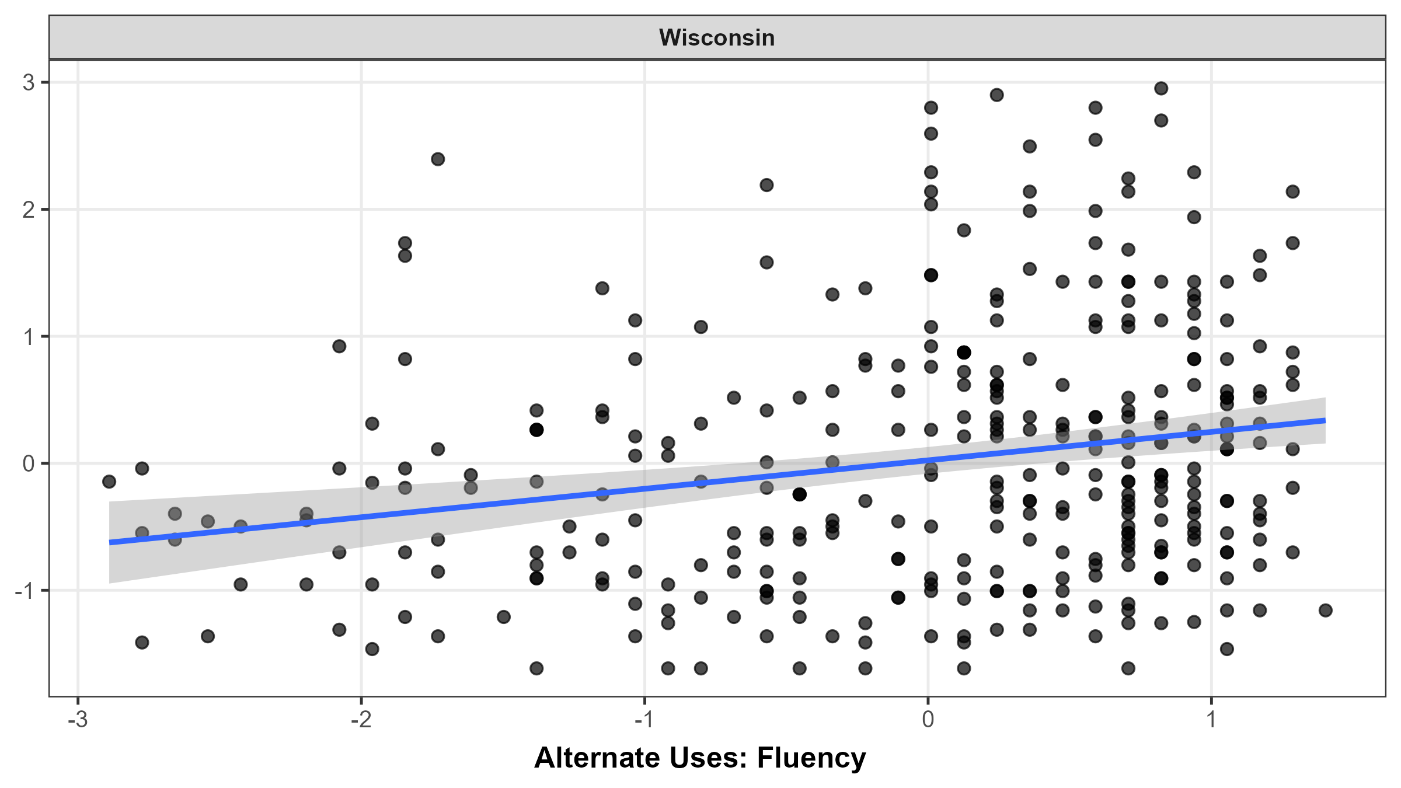


*Note*. Scatterplots between the Alternate Uses: Fluency score, and performance in the Wisconsin task. Multiple imputed datasets: 40. Sample size (after multiple imputation): 344. *Alternate Uses: Fluency*: number of valid (i.e., unusual) ideas generated in 3 minutes during the Alternate Uses Task.

Figure S3


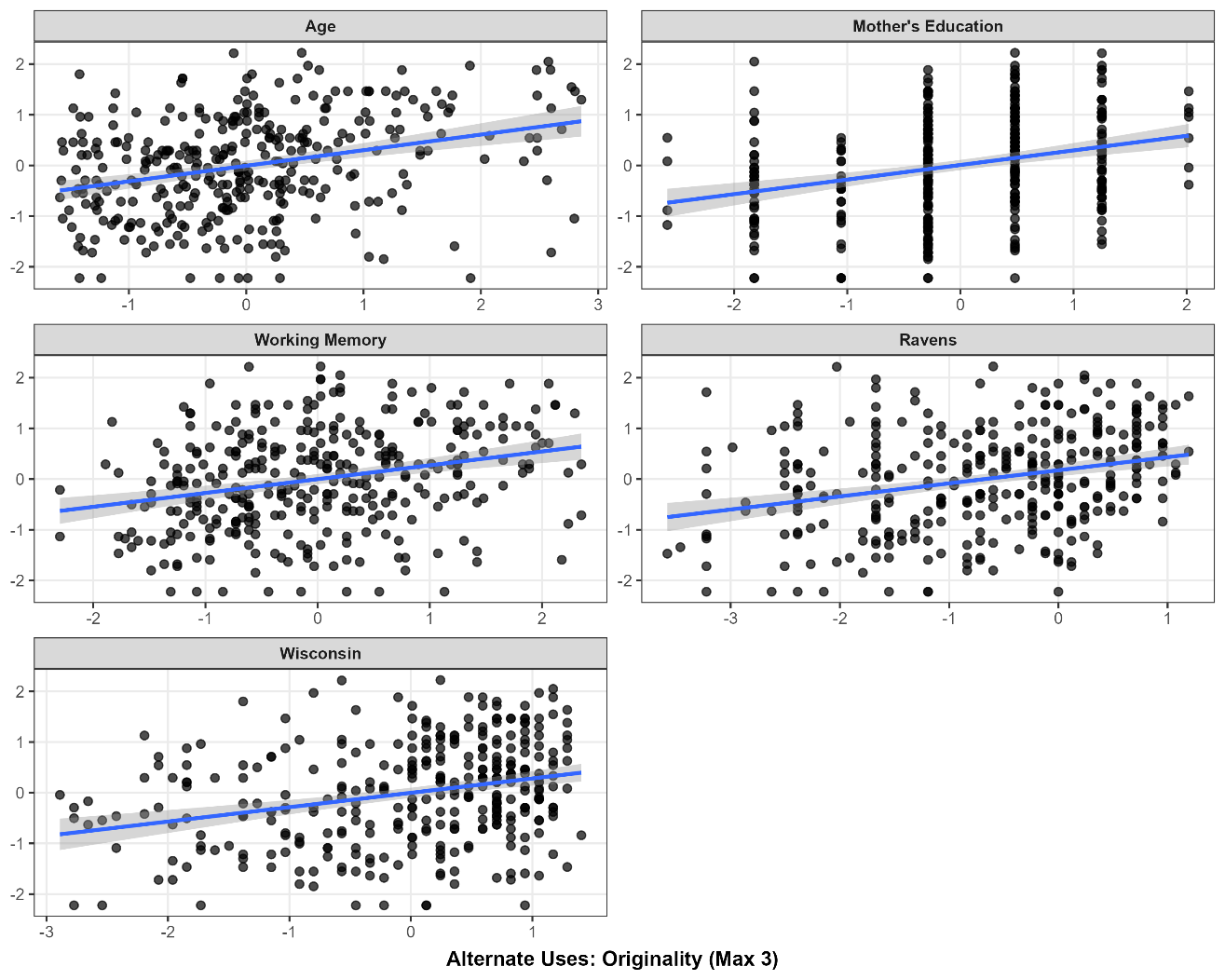


*Note*. Scatterplots between the Alternate Uses: Originality (Max 3; x-axis), and age, mother’s education scores and performance in the Working Memory, Ravens, Wisconsin tasks (y-axis). Multiple imputed datasets: 40. Sample size (after multiple imputation): 344. *Alternate Uses: Originality (Max 3):* indicate participants’ top 3 most creative responses averaged across the different Alternate Uses Task items*.*

Figure S4


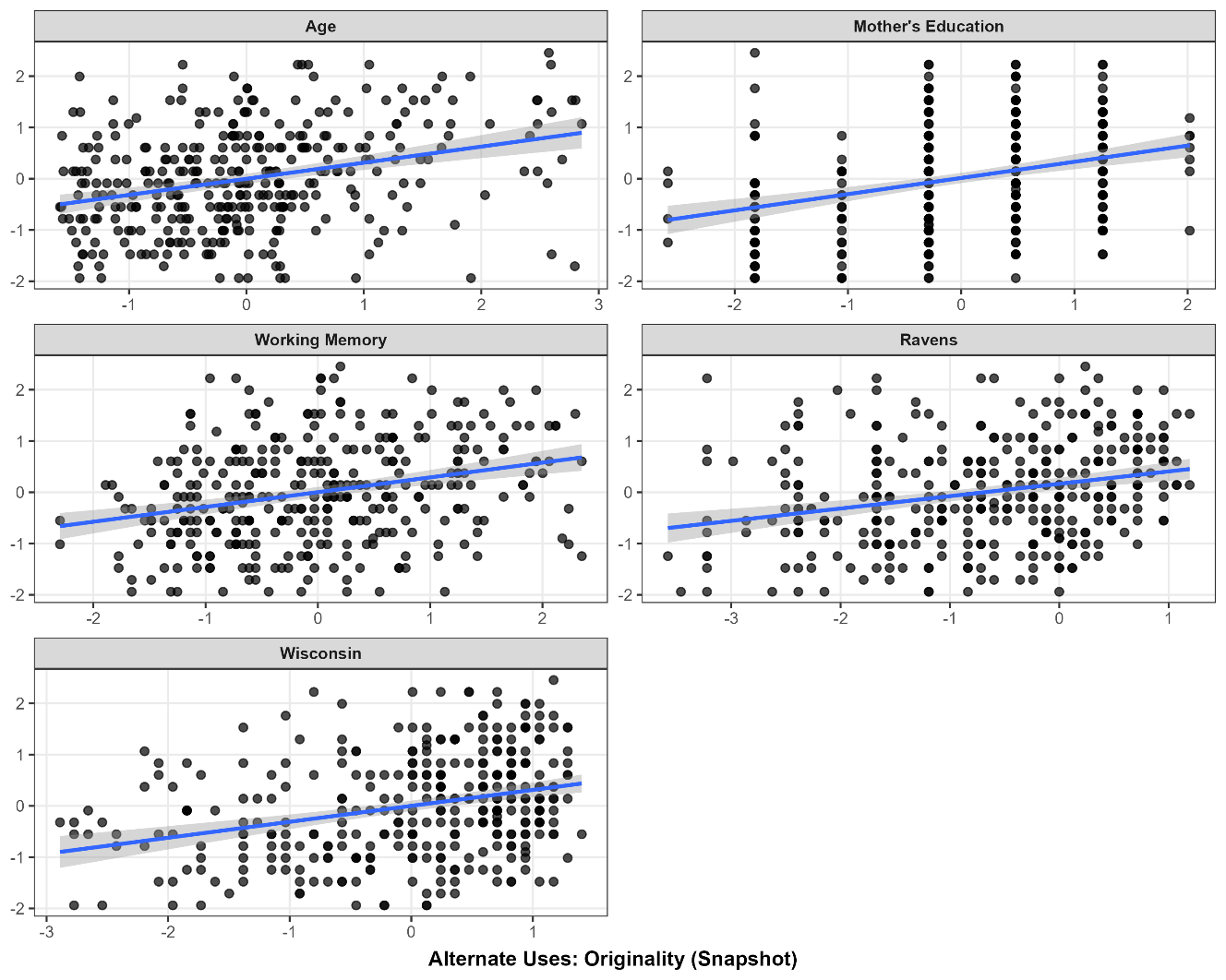


*Note*. Scatterplots between the Alternate Uses: Originality (Snapshot; x-axis), and age, mother’s education scores and performance in the Working Memory, Ravens, Wisconsin tasks (y-axis). Multiple imputed datasets: 40. Sample size (after multiple imputation): 344. *Alternate Uses: Originality (Snapshot)*: overall creative responses averaged across the different Alternate Uses Task items. **Table S2**

*Hierarchical Regression Results with Linear Mixed-Effects Models with Random Intercept for Setting.*

*Dependent variable: Originality (Max 3) performance*.

| Variable | B | p | 95% CI for B | | SE B | β | R² | ΔR² |
| --- | --- | --- | --- | --- | --- | --- | --- | --- |
|  |  |  | LL | UL |  |  |  |  |
| Step 1 |  |  |  |  |  |  | .08 |  |
| Constant | .01 | .96 | -.45 | 0.47 | 0.23 | .02 |  |  |
| Age | .18** | .00 | .06 | 0.30 | 0.06 | .18** |  |  |
| Mother's Education | .16* | .03 | .02 | 0.30 | 0.07 | .16* |  |  |
| Father's Education | .05 | .44 | -.08 | 0.19 | 0.07 | .05 |  |  |
| Step 2 |  |  |  |  |  |  | .16 | .07 |
| Constant | .10 | .56 | -.23 | .43 | .17 | .02 |  |  |
| Age | .18** | .00 | .07 | .30 | .06 | .18** |  |  |
| Mother's Education | .15* | .03 | .01 | .29 | .07 | .15* |  |  |
| Father's Education | .01 | .91 | -.13 | .14 | .07 | .01 |  |  |
| Working Memory | .10 | .06 | -.01 | .21 | .05 | .10 |  |  |
| Stroop | -.09 | .09 | -.19 | .01 | .05 | -.09 |  |  |
| Ravens | .14** | .00 | .05 | .23 | .05 | .16** |  |  |
| Step 3 |  |  |  |  |  |  | .20 | .04 |
| Constant | .06 | .72 | -.28 | .40 | .17 | .02 |  |  |
| Age | .17** | .00 | .06 | .29 | .06 | .18** |  |  |
| Mother's Education | .14* | .04 | .01 | .27 | .07 | .14* |  |  |
| Father's Education | .02 | .80 | -.12 | .15 | .07 | .02 |  |  |
| Working Memory | .07 | .23 | -.04 | .17 | .05 | .07 |  |  |
| Stroop | -.09 | .07 | -.19 | .01 | .05 | -.09 |  |  |
| Ravens | .08 | .11 | -.02 | .19 | .05 | .09 |  |  |
| Wisconsin | .14* | .02 | .03 | .26 | .06 | .14* |  |  |
| Intra Extra Shift | .01 | .93 | -.10 | .11 | .05 | .01 |  |  |
| Trails | .09 | .07 | -.01 | .19 | .05 | .09 |  |  |
| Switching | -.13* | .01 | -.22 | -.03 | .05 | -.13* |  |  |
| Note: B = fixed-effect coefficient; SE = standard error; CI = 95% confidence interval. β = standardized fixed effect computed per imputed dataset. R² values are Nakagawa marginal R². ΔR² is shown as the difference in mean marginal R² across steps. Multiple imputed datasets = 40. Sample size (after imputation): 344. *p ≤ .05. **p ≤ .01. ***p ≤ .001.  ^a^ Working Memory: CANTAB Spatial Working Memory task.  ^b^ Ravens: Raven’s Standard Progressive Matrices task.  ^c^ Wisconsin: Wisconsin Card Sorting Task.  ^d^ Intra-Extra Shift: CANTAB Intra- and Extra-Dimensional Shift Task.  ^e^ Trails: Trails Making Task A and B.  ^f^ Switching: Task Set Switching task. | | | | | | | | |
